# Association Between Dietary Regimens and Eating Disorder Susceptibility Among Adults in Riyadh, Saudi Arabia

**DOI:** 10.1002/brb3.71688

**Published:** 2026-09-10

**Authors:** Rehab A. Aldahash, Haifa Y. Alturki, Shahad G. Alonazi, Joury Dabbour, Shahad M. Aldossari, Sara Aldhfayan

**Affiliations:** ^1^ Department of Health Sciences College of Health and Rehabilitation Sciences Princess Nourah Bint Abdulrahman University Riyadh Saudi Arabia

**Keywords:** eating disorder susceptibility, dieting behavior, dietary patterns, Eating Attitudes Test (EAT‐26), Saudi Arabia

## Abstract

**Objective:**

To systematically examine the association between dietary practices and eating disorder susceptibility among adults in Riyadh, Saudi Arabia, and to determine whether specific dieting characteristics are associated with elevated eating disorder symptoms.

**Method:**

A cross‐sectional study was conducted among Saudi adults aged 18–45 years residing in Riyadh between December 2024 and August 2025. Participants were recruited through online platforms using convenience sampling. Eating disorder susceptibility was assessed using the validated Eating Attitudes Test (EAT‐26), with a score ≥ 20 indicating elevated susceptibility. Dietary behaviors during the previous 6 months were self‐reported and categorized into index‐based, macronutrient‐focused, pattern/lifestyle, and special or plant‐based diets. Multivariable logistic regression models and general linear models were used to examine associations between dietary variables and eating disorder susceptibility and symptom severity.

**Results:**

Among the 188 participants included in the primary analysis, 90 (47.9%) participants were susceptible to eating disorders. Participants who had followed any dietary regimen in the previous 6 months were significantly more likely to exhibit eating disorder susceptibility compared with non‐dieters (*p* < 0.001).

**Discussion:**

These findings suggest that recent engagement in dieting, rather than specific dietary characteristics, is associated with increased eating disorder susceptibility among adults in Riyadh. Public health efforts should emphasize balanced, evidence‐based dietary guidance and increased awareness of the potential psychological risks of unsupervised dieting. Longitudinal research is needed to clarify temporal relationships and causal pathways.

## Graphical abstract

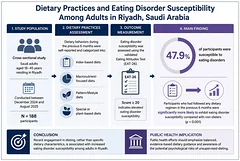

## 1 Introduction

Eating disorders (EDs) are serious mental health conditions characterized by disturbances in eating behaviors, often driven by concerns related to body weight, shape, and control over food intake (National Institute of Mental Health 2024). These disorders affect individuals across genders and age groups and are associated with substantial psychological burden and increased mortality risk (van Eeden et al. 2021). Globally, lifetime prevalence estimates suggest that anorexia nervosa affects up to 4% of females and 0.3% of males, while bulimia nervosa affects up to 3% of females and more than 1% of males (van Eeden et al. 2021). Moreover, EDs collectively affect approximately 2%–5% of individuals worldwide during their lifetime, underscoring their significance as a global public health concern (Galmiche et al. 2019; Attia and Walsh 2025).

In Saudi Arabia, disordered eating behaviors appear to be particularly prevalent. Previous studies have reported that approximately 25%–35% of adults, especially females, exhibit elevated ED symptoms or unhealthy eating attitudes (Waseem and Ahmad 2018; Alwosaifer et al. 2018; Abd El‐Azeem Taha et al. 2018). This high prevalence may partly reflect the rapid sociocultural changes occurring in Saudi Arabia, including the increasing internalization of Western values and concerns related to body image, both of which have been associated with ED pathology among Saudi populations (Melisse et al. 2022; Alshebali et al. 2020). Previous research in the Arab world has identified body dissatisfaction, desire for thinness, dieting behaviors, media use, Western influences, and obesity as factors associated with increased ED‐related variables, highlighting the importance of culturally informed approaches for understanding the scope and determinants of ED susceptibility in the Saudi population (Melisse et al. 2020).

Dietary restraint refers to the cognitive and intentional effort to limit food intake, typically for weight control or body weight management purposes. This construct reflects the intention to restrict eating and does not necessarily indicate a reduction in caloric intake (Coffino et al. 2016; Hagerman et al. 2021). The relationship between dietary restraint and ED risk is complex. Although dietary restraint has historically been considered a risk factor for eating pathology, emerging evidence suggests that it may also support healthy weight management and is not associated with increased eating pathology when practiced within well‐validated weight management programs. In contrast, engagement in dieting practices has been associated with greater ED psychopathology, although these associations may vary according to the type of dieting practice and population studied (Schaumberg et al. 2016; Fan et al. 2024). Empirical evidence further indicates that a history of engaging in more restrictive or effort‐intensive dietary practices is commonly observed among individuals with EDs and may be associated with greater symptom severity (Shahan et al. 2020). Longitudinal research further supports the temporal relevance of this relationship. In a landmark prospective study among adolescent females, individuals who reported dieting exhibited an approximately eightfold increased risk of developing an ED compared with their non‐dieting peers (Patton et al. 1990).

Within the Saudi context, popular diets such as ketogenic, intermittent fasting, gluten‐free, and calorie‐restricted regimens are increasingly adopted for weight loss purposes; however, many individuals experience difficulties sustaining adherence over time (Alhusseini et al. 2024). Additionally, vegetarian dietary patterns have been increasingly reported among adults in Saudi Arabia, often motivated by health‐related, ethical, or environmental considerations (Azhar et al. 2023), consistent with broader international trends (Sergentanis et al. 2020). However, empirical evidence examining how engagement in these specific dietary regimens, along with dieting characteristics such as duration, perceived progress, adherence challenges, and sources of dietary information, relates to ED susceptibility among adults in Saudi Arabia remains limited.

Therefore, this study aimed to systematically assess ED susceptibility among adults aged 18–45 years in Riyadh, Saudi Arabia, and to examine its association with recent engagement in dietary regimens. Specifically, the study investigated whether diet type, duration of adherence, perceived dietary progress, adherence challenges, and sources of dietary information were associated with elevated ED susceptibility. By addressing this gap, the study seeks to inform culturally relevant prevention strategies and promote safer, evidence‐based dietary practices within the Saudi population.

## 2 Methodology

### 2.1 Study Design and Participants

A cross‐sectional study was conducted among Saudi adults aged 18–45 years residing in Riyadh, Saudi Arabia, between December 2024 and August 2025. Participants were recruited using non‐probability convenience sampling through online platforms, including WhatsApp, X, and Telegram. Women and men within this age range were included to maintain hormonal and metabolic consistency, as adults over 45 years may undergo menopausal or age‐related physiological changes that increase the risk of disordered eating behaviors (Vincent et al. 2024). Pregnant and breastfeeding women were excluded due to hormonal and psychological changes that may influence eating behaviors and confound the association between dietary practices and ED susceptibility (Martínez‐Olcina et al. 2020). Individuals reporting a prior clinical diagnosis of an ED were also excluded.

A total of 507 responses were received. Of these, 188 participants with complete data were included in the primary analytical models. All 507 respondents were retained for analyses examining the binary exposure of having followed any dietary regimen in the previous 6 months.

### 2.2 Data Collection and Questionnaire

Data were collected using a self‐administered online questionnaire. The survey collected sociodemographic information, including age, gender, education level, weight, and height, followed by a screening question asking whether participants had followed any dietary regimen in the previous 6 months. The recall period was restricted to 6 months to improve the accuracy of self‐reported dietary behaviors and minimize recall bias. Participants who answered “yes” proceeded to the dietary practices section, while those who answered “no” were directed directly to the eating attitudes assessment.

Dietary practices were assessed using a study‐specific questionnaire, in which participants could select all dietary regimens they had followed over the previous 6 months. The study‐specific dietary practices questionnaire consisted of closed‐ended multiple‐choice items, including single‐response and multiple‐response questions across five domains: dietary regimen type, adherence duration, perceived progress, adherence challenges, and sources of dietary information. The questionnaire was developed by the research team based on relevant literature and adapted to suit the Saudi context. Content validity was assessed by five experts in nutrition and public health. Due to sample size considerations and overlap between dietary patterns, reported diets were grouped into four analytical categories: index‐based diets, defined as dietary patterns commonly assessed using validated diet quality or adherence scoring systems, including DASH and Mediterranean diets (Brlek and Gregorič 2022); macronutrient‐focused diets (ketogenic, low‐carbohydrate, and low‐fat) (Feng et al. 2025); pattern/lifestyle diets (intermittent fasting and calorie restriction) (Wu et al. 2025); and special or plant‐based diets (vegetarian/vegan, gluten‐free) (Hargreaves et al. 2023; El Khoury et al. 2018).  Adherence duration in the previous 6 months was categorized as < 1 month, 1–3 months, or 4–6 months. Subsequently, eating attitudes were assessed using the validated Eating Attitudes Test (EAT‐26) to evaluate susceptibility (Garner et al. 1982). The EAT‐26 consists of 26 items rated on a 6‐point Likert scale. For items 1–25, responses are scored as follows: “Always” = 3 points, “Usually” = 2 points, “Often” = 1 point, while “Sometimes,” “Rarely,” and “Never” are scored as 0. Item 26 is reverse scored, with “Never” = 3 points, “Rarely” = 2 points, “Sometimes” = 1 point, and “Often,” “Usually,” and “Always” scored as 0. Total scores are calculated by summing all item scores; a cutoff of ≥ 20 indicates elevated ED susceptibility and the need for further clinical evaluation (Garner et al. 1982).

### 2.3 Validity and Reliability

The dietary practices section underwent content validation by five experts in nutrition and public health, whose feedback was incorporated before data collection. The EAT‐26 was translated into Arabic using a forward–backward translation procedure to ensure linguistic and conceptual equivalence, followed by expert review to confirm cultural appropriateness. The internal consistency of the Arabic EAT‐26 demonstrated good reliability in this sample (Cronbach's *α* = 0.842; 95% CI: 0.842–0.843). Formal pilot testing was not conducted; however, expert review confirmed clarity and readiness for data collection.

### 2.4 Statistical Analysis

All statistical analyses were conducted using IBM SPSS Statistics (Version 25). ED susceptibility was defined according to the EAT‐26 criteria and coded as a binary variable, with scores ≥ 20 indicating susceptibility and scores < 20 indicating non‐susceptibility. Continuous variables were summarized as means and standard deviations, and categorical variables were summarized as frequencies and percentages. Associations between participant characteristics and eating‐disorder susceptibility were examined using multivariable binary logistic regression. This model included age group, gender, BMI category, and education level as predictors, and odds ratios (ORs) with 95% confidence intervals (CIs) were reported for each comparison. Dietary practice variables, including number of diets, diet type, diet duration, perceived progress, adherence challenges, and information sources, were each examined in separate binary logistic regression models, with ORs, 95% CIs, and Wald *p*‐values reported. To further evaluate the relationship between dieting behavior and eating‐disorder symptoms, a general linear model (GLM) was used to compare continuous EAT‐26 scores across diet‐duration categories, with the < 1‐month group serving as the reference. Mean differences, their associated CIs, and significance levels were extracted directly from the model. In addition, a multivariable logistic regression model was constructed to assess whether the number and type of dietary information sources were associated with susceptibility. This model simultaneously included the number of sources used and each source category (family/friends, online media, health professionals, and books/printed publications). Finally, the association between having followed any diet in the previous 6 months and susceptibility was evaluated using a chi‐square test, as the dataset structure allowed only a binary exposure for this variable. All analyses were two‐tailed, and a *p*‐value < 0.05 was considered statistically significant. Logistic regression models were also assessed for convergence and sparse‐cell issues, and interpretation emphasized effect sizes alongside statistical significance.

### 2.5 Ethical Considerations

This study was approved by the Institutional Review Board (IRB) of Princess Nourah Bint Abdulrahman University (Approval Number: 24–0931). Before participation, informed consent was obtained via the online questionnaire, which clearly explained the study's purpose and objectives. Participation was entirely voluntary, and no personally identifiable information was collected. Participants were informed of their right to withdraw at any stage, and a contact method was provided for inquiries. To ensure data confidentiality, all responses were anonymized and numerically coded. Participants did not receive any financial compensation for participation in this study. The copyright holder obtained permission to use the EAT‐26 scale from Dr. David M. Garner.

## 3 Results

**FIGURE 1:**
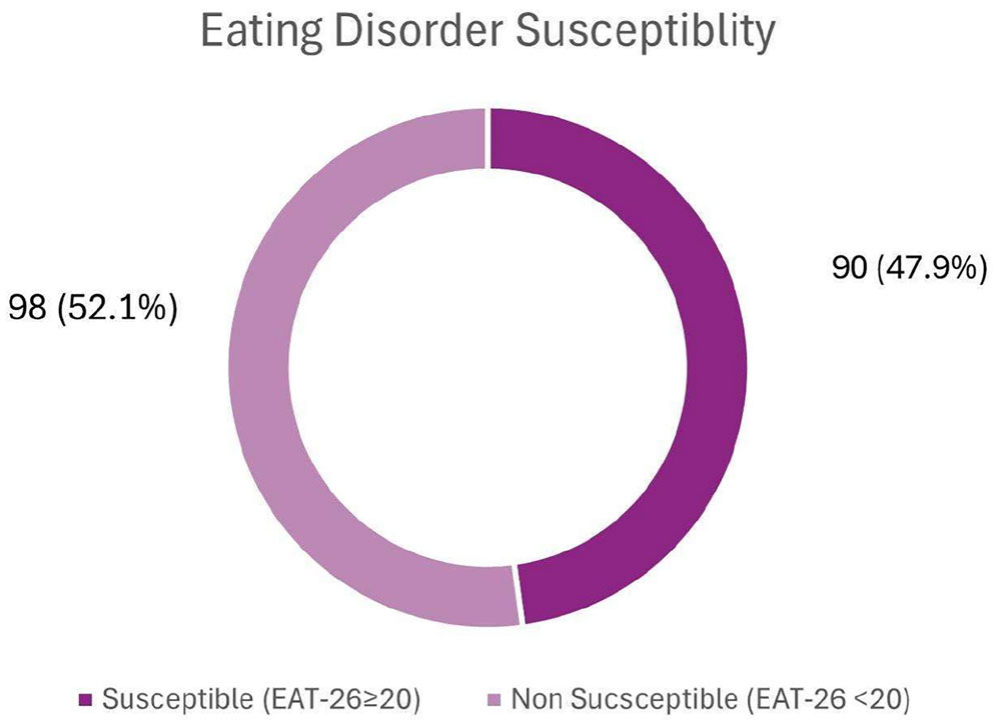
Prevalence of eating disorder susceptibility among study participants (*n* = 90). Eating disorder susceptibility was determined using the Eating Attitudes Test (EAT‐26). Participants with scores ≥ 20 were classified as susceptible, whereas those with scores < 20 were classified as non‐susceptible. Values represent the number and percentage of participants in each group.

A total of 188 participants were included in the analysis, of whom 90 (47.9%) met the threshold for eating‐disorder susceptibility based on the EAT‐26, while 98 (52.1%) were classified as non‐susceptible. This distribution is illustrated in Figure 1. In the multivariable logistic regression model examining baseline demographic and clinical characteristics (Table 1), none of the predictors were significantly associated with susceptibility. Compared with participants aged 18–24 years, neither those aged 25–35 nor those aged 36–45 showed significantly different odds of susceptibility. Gender was not related to susceptibility, and BMI category also demonstrated no significant associations. Education level displayed wide CIs due to sparse responses in some categories, and no meaningful pattern emerged. Overall, demographic characteristics did not appear to influence the likelihood of elevated eating‐disorder symptoms.

**TABLE 1:** Baseline demographic and clinical characteristics.

	Total	Susceptible	Non‐susceptible	OR	95%CI	*p*‐value
	(*n* = 188)	(*n* = 90)	(*n* = 98)			
Age 18–24 25–35 36–45	110 (58.5) 43 (22.9) 35 (18.6)	55 (61.1) 21 (23.3) 14 (15.6)	55 (56.1) 22 (22.4) 21 (21.4)	1 1.939 1.560	— 0.818–4.596 0.597–4.079	— 0.133 0.364
Gender Male Female	35 (18.6) 153 (81.4)	17 (18.9) 73 (81.1)	18 (18.4) 80 (81.6)	1 1.121	— 0.499–2.518	— 0.782
BMI Underweight Normal weight Overweight/obese	8 (4.3) 78 (41.5) 102 (54.3)	4 (4.4) 40 (44.4) 46 (51.1)	4 (4.1) 38 (38.8) 56 (57.1)	1 1.160 1.265	— 0.262–5.137 0.687–2.329	— 0.845 0.450
Education level Secondary education or less Diploma Bachelor's Postgraduate	32 (17) 11 (5.9) 128 (68.1) 17 (9)	15 (16.7) 6 (6.7) 59 (65.6) 10 (11.1)	17 (17.3) 5 (5.1) 69 (70.4) 7 (7.1)	1 0.618 0.840 0.599	— 0.188–2.029 0.182–3.880 0.214–1.671	— 0.427 0.823 0.327
Diet type^a^ Index‐based Macronutrient Pattern/lifestyle Special/plant‐based	45 (23.9) 127 (67.5) 169 (89.9) 51 (27.1)	19 (21.1) 59 (65.5) 84 (93.3) 24 (26.7)	26 (26.5) 68 (69.4) 85 (86.7) 27 (27.6)	1 0.820 0.967 1.085	— 0.365–1.840 0.502–1.862 0.571–2.061	— 0.630 0.920 0.803
Number of diets followed^b^	3.38 ± 2.21	3.50 ± 2.26	3.27 ± 2.16	−0.224	−0.860 to 0.411	0.487

All values presented in number (percentage).

^a^Multiple answers were allowed.

^b^ Mean ± SD

]

**FIGURE 2:**
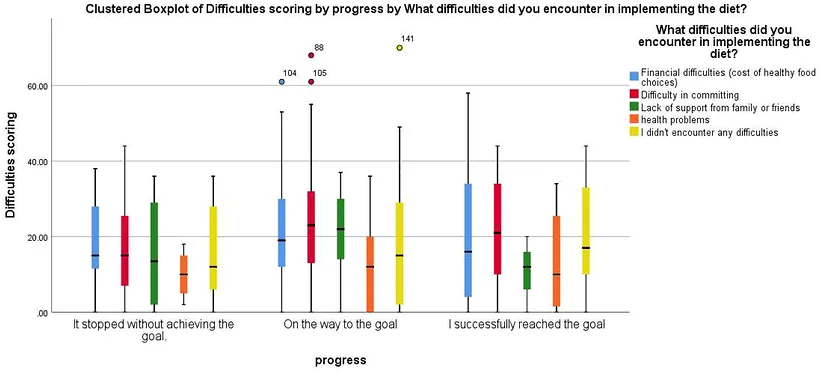
Variation in difficulty scores according to perceived diet progress and reported diet‐related barriers (multiple responses allowed). Boxplots display the median, interquartile range, and minimum and maximum values, with circles representing outliers. Difficulty scores are shown separately for each perceived progress category and barrier type.

Dietary behaviors were examined next (Table 2; Figure 2). The number of diets followed did not differ significantly between susceptible and non‐susceptible groups and showed no association with susceptibility in regression analysis. Diet type also failed to predict susceptibility, with macronutrient‐focused, pattern/lifestyle, and special/plant‐based diets all demonstrating ORs close to unity compared with index‐based diets. Diet duration similarly did not predict susceptibility. Participants who had been dieting for 1–3 months or 4–6 months showed ORs comparable to those dieting for less than a month. Perceived dietary progress was likewise unrelated to susceptibility. Whether participants had stopped without achieving their goal, were still working toward it, or had successfully reached it, the odds of susceptibility did not meaningfully differ. Self‐reported adherence challenges such as financial limitations, commitment difficulty, lack of social support, or health problems were not associated with eating‐disorder susceptibility, and none of the challenge categories reached statistical significance. Figure 2 demonstrates the distribution of difficulty scores across progress groups and challenge types, highlighting variability in subjective burden but no discernible pattern linked to susceptibility.

**TABLE 2:** Association between dietary practices and eating disorder susceptibility.

	Susceptible	Non‐susceptible	Effect size	95%CI	*p*‐value
	(*n* = 90)	(*n* = 98)			
Number of diets followed^a^	3.50 ± 2.26	3.27 ± 2.16	−0.224	−0.860 to 0.411	0.487
Diet type^b^ Index‐based Macronutrient Pattern/lifestyle Special/plant‐based	19 (21.1) 59 (65.5) 84 (93.3) 24 (26.7)	26 (26.5) 68 (69.4) 85 (86.7) 27 (27.6)	1 0.820 0.967 1.085	— 0.365–1.840 0.502–1.862 0.571–2.061	— 0.630 0.920 0.803
Diet duration^b^ Less than a month From 1 to 3 months From 4 to 6 months	44 (48.9) 68 (75.5) 74 (82.2)	55 (56.1) 77 (78.6) 74 (75.5)	1 0.831 0.873	— 0.493–1.401 0.551–1.383	— 0.487 0.562
Perceived diet progress It stopped without achieving the goal On the way to the goal I successfully reached the goal	12 (13.3) 59 (65.2) 19 (21.1)	26 (26.5) 52 (53.1) 20 (20.4)	1 0.486 1.194	1 0.192–1.230 0.575–2.479	— 0.128 0.634
Adherence challenges^b^ Financial difficulties Difficulty in committing Lack of support from family Health problems I didn't encounter any difficulties	32 (35.6) 64 (71.1) 12 (13.3) 4 (4.44) 18 (20)	38 (38.8) 64 (65.3) 11 (11.2) 13 (13.3) 25 (25.6)	1 1.170 1.389 1.515 0.427	— 0.543–2.518 0.691–2.791 0.547–4.194 0.120–1.528	— 0.689 0.356 0.424 0.191
Sources of dietary information^b^ A family member or friend Online sources Health professional Books or printed publications	20 (22.2) 58 (64.4) 31 (34.4) 8 (8.9)	24 (24.5) 68 (69.4) 34 (34.7) 5 (5.1)	1 0.521 0.533 0.570	— 0.147–1.846 0.165–1.719 0.168–1.928	— 0.312 0.292 0.366

*Note*: All values presented in number (percentage) unless indicated otherwise.

^a^ mean ± SD

^b^ Multiple answers were allowed.

Continuous EAT‐26 scores were also compared across diet‐duration using a linear regression (Table 3). Across all parameterizations of diet duration as continuous (months), categorical (< 1, 1–3, 3–6 months), and ordinal for trend, there was no significant association with EAT‐26 scores. When treated as a continuous variable, diet duration showed no meaningful effect (*B* = 0.14, 95% CI: −0.82 to 1.09, *p* = 0.778). Categorically, neither the 1–3 month group (*B* = 0.24, 95% CI: −3.94 to 4.42, *p* = 0.910) nor the 3–6 month group (*B* = 0.59, 95% CI: −3.58 to 4.75, *p* = 0.781) differed from the < 1 month reference group. An ordinal trend model likewise showed no dose–response relationship (*B* = 0.30, 95% CI: −1.77 to 2.36, *p* = 0.776). All models explained virtually no variance in EAT‐26 scores (*R* ^2^< 0.001).

**TABLE 3:** Linear regression models examining associations between diet duration and EAT‐26 scores.

	*B*	95% CI	*p*‐value
Diet duration, months	0.14	−0.82 to 1.09	0.778
Diet duration			
Less than a month From 1 to 3 months From 4 to 6 months	1	—	—
	0.24	−3.94 to 4.42	0.910
	0.59	−3.58 to 4.75	0.781
Diet duration, ordinal	0.30	−1.77 to 2.36	0.776

A multivariable logistic regression assessing the number and type of dietary information sources (Table 4) showed no significant associations with susceptibility. The number of sources used was not related to higher or lower odds of susceptibility, and none of the individual source categories (family/friends, online sources, health professionals, or printed publications) showed significant effects. CIs were broad, reflecting heterogeneity in source usage and limited statistical precision.

**TABLE 4:** Association between sources of dietary information and eating‐disorder susceptibility.

	Susceptible	Non‐susceptible	Effect size	95% CI	*p*‐value
	(*n* = 90)	(*n* = 98)			
Number of sources used^a^	1.25 ± 0.52	1.36 ± 0.58	0.508	0.035–7.385	0.620
Sources of dietary information^b^ A family member or friend Online sources Health professional Books or printed publications	20 (22.2) 58 (64.4) 31 (34.4) 8 (8.9)	24 (24.5) 68 (69.4) 34 (34.7) 5 (5.1)	1 0.521 0.533 0.570	— 0.147–1.846 0.165–1.719 0.168–1.928	‐ 0.312 0.292 0.366

*Note*: All values presented in number (percentage) unless indicated otherwise.

^a^ mean ± SD

^b^ Multiple answers were allowed.

The only variable showing a significant association with susceptibility was whether participants had followed a diet in the past 6 months (Table 5). Participants who had recently been dieting were significantly more likely to meet the EAT‐26 threshold for susceptibility than those who had not (56.6% vs. 28.2%), a difference confirmed by chi‐square testing (*p* < 0.001).

**TABLE 5:** Association between following any dietary regimen and susceptibility to eating disorders (EAT‐26).

	Susceptible	Non‐susceptible	*p*‐value
	(*n* = 159)	(*n* = 348)	
Followed any diet in the past 6 months	90 (56.6)	98 (28.2)	<0.001
Did not follow any diet in the past 6 months	69 (43.4)	250 (71.8)	

*Note*: All values presented in number (percentage).

## 4 Discussion

This study examined ED susceptibility among adults in Riyadh and its association with various dietary practices. Nearly half of the sample (47.9%) met the EAT‐26 threshold for susceptibility, indicating a substantial burden of elevated disordered eating attitudes. Although this prevalence is higher than previously reported Saudi estimates, it remains comparable to earlier findings, including 36.8% among female university students in Riyadh (Albrahim et al. 2019), 32.1% among medical students in Jeddah (Ghamri et al. 2022), and 29.4% in Dammam (Alwosaifer et al. 2018). A similar international study from Bangladesh reported a prevalence of 23% (Banna et al. 2021). Together, these findings underscore a growing concern regarding ED susceptibility in the region. Sociocultural shifts observed across the Arab world, including increased exposure to Western beauty ideals and social media, may help explain elevated levels of body dissatisfaction and disordered eating attitudes and are likely relevant to the Saudi context (Melisse et al. 2020).

Although age and gender were not statistically significant predictors in multivariable analysis, the demographic distribution of the sample remains noteworthy. Most participants were young adults aged 18–24 years, and females constituted the majority. Previous Saudi studies have consistently reported higher susceptibility among females, including significantly elevated odds in samples from Jeddah and Taiba University (Ghamri et al. 2022; Alhazmi and Al Johani 2019). Findings from Majmaah University further suggest that female students are more psychologically preoccupied with food and calorie intake (Loni et al. 2022), supporting the role of sociocultural pressures related to body image and thinness. BMI was not significantly associated with susceptibility in the present study. This contrasts with several Saudi studies reporting higher ED risk or elevated EAT‐26 scores in relation to body weight and weight‐related factors, including overweight or obesity in some populations (Ghamri et al. 2022; Alhazmi and Al Johani 2019), as well as heightened ED concerns linked to sociocultural pressures surrounding weight and appearance (Loni et al. 2022; Alwosaifer et al. 2018). These discrepancies may reflect differences in sample composition, measurement approaches, or contextual influences, highlighting the complex and nonlinear relationship between BMI and ED susceptibility.

Across all dietary variables examined, the only factor significantly associated with susceptibility was having followed any diet within the past 6 months, as defined in the study questionnaire. More than half of recent dieters (56.6%) met the EAT‐26 threshold, compared with 28.2% of non‐dieters (*p* < 0.001). This finding is consistent with prevention research, which indicates that reductions in dieting attempts are associated with decreases in ED symptoms in intervention settings (Stice et al. 2009). Taken together, these findings suggest that recent engagement in dieting, rather than specific dietary characteristics, may be relevant when considering ED susceptibility.

Diet type did not predict susceptibility despite descriptive differences in popularity and gender distribution. Macronutrient‐focused, pattern/lifestyle, and plant‐based or special diets all demonstrated ORs close to unity. Pattern/lifestyle diets, such as calorie restriction and intermittent fasting, were the most commonly adopted, reflecting prevailing dietary trends (Alsaleh et al. 2024). This contrasts with earlier findings reporting associations between intermittent fasting and ED behaviors and psychopathology (Ganson et al. 2022). In the current study, however, neither diet type nor the number of diets followed showed a significant association with susceptibility. Within the 6‐month observation window used in this study, no association was observed between diet duration and ED susceptibility. However, these findings should be interpreted cautiously because the study assessed dieting only during the previous 6 months. Individuals who have engaged in dieting for years may have been classified similarly to recent dieters, potentially obscuring longer term associations. Likewise, no significant associations were observed for perceived progress, and adherence challenges were also unrelated to susceptibility or continuous EAT‐26 scores, with no evidence of a dose–response relationship. These findings support the view that ED susceptibility is multifactorial and not driven by isolated dietary behaviors (Barakat et al. 2023). Additionally, because of the cross‐sectional design, the direction of the observed association cannot be determined. Individuals with elevated ED symptoms may be more likely to initiate dieting behaviors, just as dieting itself may contribute to ED susceptibility.

No significant associations were observed between susceptibility and the number or type of dietary information sources used. While minor descriptive differences were noted, such as slightly greater reliance on online sources among susceptible participants, these did not reach statistical significance. This is consistent with literature highlighting that vulnerability to ED symptoms is influenced by psychological and sociocultural factors, including body dissatisfaction, weight concern, and exposure to media or dietary information (Melisse et al. 2020; Georgiou et al. 2025).

### 4.1 Strengths and Limitations

This study has several strengths. It provides detailed characterization of contemporary dietary practices in association with ED susceptibility using a validated screening tool (EAT‐26). The inclusion of multiple diet types, adherence duration, perceived progress, and information sources offers a comprehensive behavioral perspective. Additionally, the use of standardized scoring procedures and multivariable analytical models strengthens internal validity and allows for robust assessment of associations. However, several limitations should be acknowledged. The cross‐sectional design precludes causal inference between dietary practices and ED susceptibility. The use of non‐probability convenience sampling and self‐reported data may limit generalizability and introduce selection and reporting bias. Dietary behaviors and anthropometric measures were self‐reported and therefore subject to recall bias. Restricting dietary assessment to the previous 6 months reduced recall bias but may not have captured longer term dieting behaviors. Therefore, individuals with chronic dieting histories may have been misclassified. Additionally, as the study was conducted entirely online without bot‐prevention measures, the possibility of nonhuman responses cannot be excluded. Finally, although the EAT‐26 is a validated screening instrument, it does not provide a clinical diagnosis, and the relatively small analytical sample may have limited statistical power to detect modest associations.

## 5 Conclusion

The findings of this study highlight the importance of considering dieting behaviors as a relevant public health concern in relation to ED susceptibility among adults in Saudi Arabia. These results underscore the need for increased awareness regarding the potential psychological risks associated with self‐directed dietary practices. Public health initiatives should emphasize balanced, evidence‐based dietary guidance and promote access to qualified nutrition professionals. Future research using longitudinal designs and representative samples is recommended to clarify temporal relationships and inform targeted prevention strategies.

## Author Contributions

**Rehab A. Aldahash**: conceptualization, investigation, funding acquisition, methodology, validation, Writing–review and editing, project administration, supervision, data curation. **Haifa Y. Alturki**: conceptualization, investigation, writing ‐ original draft, methodology, validation, visualization, writing ‐ review and editing, formal analysis, data curation. **Shahad G. Alonazi**: conceptualization, investigation, writing ‐ original draft, methodology, validation, data curation. **Shahad M. Aldossari**: conceptualization, investigation, methodology, validation, data curation. **Joury Dabbour**: conceptualization, investigation, writing ‐ original draft, methodology, validation, visualization, data curation. **Sara Aldhfayan**: conceptualization, investigation, methodology, validation, data curation.

## Funding

This research was funded by Princess Nourah bint Abdulrahman University Researchers Supporting Project number (PNURSP2026R533), Princess Nourah bint Abdulrahman University, Riyadh, Saudi Arabia.

## Ethics Statement

The study was approved by the Institutional Review Board of Princess Nourah bint Abdulrahman University (Approval# 24–0931).

## Consent

Informed consent was obtained from all participants involved in the study.

## Conflicts of Interest

The authors declare no conflicts of interest.

## Data Availability

The datasets generated and analyzed during the current study are available from the corresponding author on reasonable request.
